# COVID-19 infection in known epileptic and non-epileptic children: what is the place of chloroquine sulfate? (a case report)

**DOI:** 10.11604/pamj.2020.37.177.26066

**Published:** 2020-10-22

**Authors:** Hugues Ghislain Atakla, Aïtchéou Cadnel Wilfried Houedenou Noudohounsi, Lounceny Fatoumata Barry, Mahugnon Maurel Ulrich Dénis Noudohounsi, Lauréano Déo-gratias Legba, Ibrahima Sory Souare, Fatoumata Kaba, Dismand Stephan Houinato

**Affiliations:** 1 Neurology Department, University Hospital Center Hubert Koutoukou MAGA, Cotonou, Benin,; 2 Intensive Care Unit, University Hospital Center Hubert Koutoukou MAGA, Cotonou, Benin,; 3 Neurosurgery Department, Ignace Deen University Hospital Center, Conakry, Guinea,; 4 Public Health Physician, Research Project Manager, Brazzaville, Congo

**Keywords:** Epileptic seizures, coronavirus, epileptic malaise, chloroquine sulfate

## Abstract

The coronavirus 19 (COVID-19) disease, which was declared in China in December 2019, very early on became a pandemic, claiming more than 28 million victims worldwide to date. Its impact on the central nervous system is still poorly understood. The objective of this work is to assess the involvement of severe acute respiratory syndrome coronavirus-2 (SARS-CoV-2) in the aggravation of seizures in children known to have epilepsy and in the epileptogenesis of children hitherto seizure-free. Prior to conducting this work, we had obtained informed consent from patients and parents. We report the cases of three (3) patients, one known epileptic and the other two apparently healthy, who presented a febrile seizure in a context of COVID-19 infection. The aggravation of the epileptic seizure was indicative of a SARS-CoV-2 infection in the first patient, while the seizure occurred after induction of chloroquine sulfate treatment in the 2 other patients. Although our current concern is to limit the spread of the disease to COVID-19, it is crucial to address its possible complications. Notably, the worsening of seizures in children with epilepsy and the occurrence of first seizures in children without epilepsy following drug treatment. Equipping our COVID-19 patient management facilities with electroencephalogram (EEG) equipment could facilitate continuous electroencephalographic monitoring of children for proper management.

## Introduction

The coronavirus 19 (COVID-19) disease was first recognized in Hubei Province in China in December 2019, became a pandemic early on, killing 28,219,714 people worldwide [1]. It has thus become the focus of concern for health actors around the world. The virus targets the respiratory system, but it also has neuroinvasive capabilities [2]. There is currently clinical evidence that patients with COVID-19 may experience symptoms similar to those of an intracranial infection such as headache, dizziness, sudden loss of sense of smell and taste, altered mental status and seizures [3, 4]. However, epileptic seizures have not been directly reported as part of COVID-19 apart from patients with previously known brain injury or epilepsy. Epileptic seizures have several origins, with infectious causes most commonly reported in children in Africa. Our current understanding of the neurological involvement of the SARS-CoV-2 pathogen COVID-19 in patients with epileptic seizures remains very limited. Furthermore, acute symptomatic seizures are possible in patients with COVID-19. These seizures are likely to be multifactorial in origin, including cortical irritation due to rupture of the blood-brain barrier, precipitated by the cytokine reaction in viral infection [5]. Although chloroquine and hydroxychloroquine treatment is controversial worldwide, these molecules are in great demand for the treatment of COVID-19 in Africa. However, chloroquine at an effective dose (10-50 mg/kg) is susceptible to epileptic seizures by inhibiting GABAergic neurotransmission [6]. But does the SARS-CoV-2 infection cause the first seizure in some subjects; does the infection cause recurrence of seizures in the known epileptic subject? Is chloroquine treatment judicious in this context? In this report, we describe three patients, one known epileptic and the other two apparently healthy, who presented a febrile seizure in a context of COVID-19 infection.

## Patient and observation

**Figure 1:**
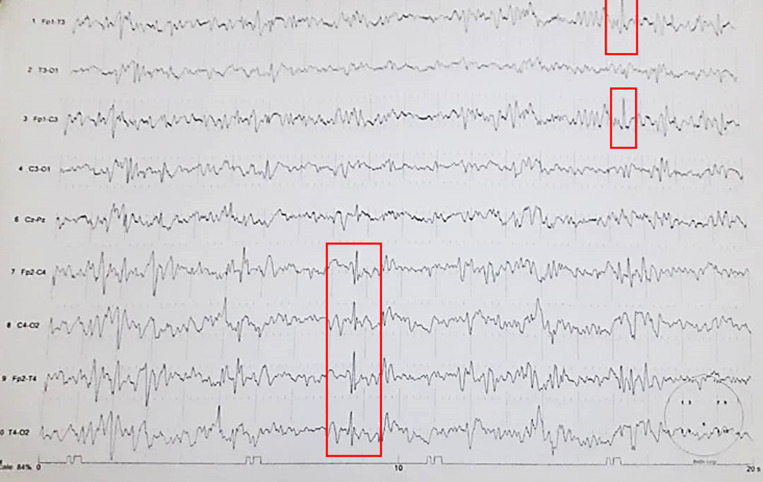
trace of 4-5Hz theta activity, ample, symmetrical, bilateral associated with epileptogenic graphoelements of diffuse point type accentuated in the right hemisphere, suggesting multifocal epilepsy

**Case N°1:** 14 years old patient admitted to the emergency room for loss of consciousness, recurrent tonic-clonic seizures and post critical confusion for 4 hours prior to admission. The hetero anamnesis collected from his family reveals that the patient has been known to be epileptic since the age of 9 years, on sodium valproate regularly followed with the frequency of one seizure in 16 months. The parents report that the patient had 9 seizures without a return of consciousness within 4 hours before being admitted to the emergency room. This unprecedented state of seizure is believed to be preceded by a 13-day-old flu-like syndrome, which was treated unsuccessfully with: acetaminophen and ascorbic acid before admission. There has been no known exposure to COVID-19 and has no known vice or allergy. In addition, the clinical examination found a temperature (T°) of 38.6°C; tachycardia at 117 beats/minute, oxygen saturation of 86%; blood pressure of 90/50mmHg. The neurological examination was not further investigated due to the clinical condition of the patient. He was transferred to the intensive care unit, put on oxygen and antipyretic medication and vascular filling with crystalloids (30ml/kg), while waiting for the result of the metabolic workup, which proved normal. The biological investigation revealed some disorders including: a non-specific inflammatory syndrome with a sedimentation rate (SV) at 62mm/h; a C-reactive protein (CRP) at 36mg/l and white blood cells > 12000/mm^3^. While in hospital, the patient presented with repeated paroxysmal seizures within a short period of time, without a return to normal consciousness between seizures. The diagnosis of status with septic shock was made and the patient strictly followed the protocol for the management of status epilepticus (Clonazepam 1ml + Phenobarbital 60mg/kg by slow infusion + Thiopental 5mg/kg as a bolus). He did a brain computed tomography (CT) scan which did not reveal any specifics. The intercritical EEG tracing performed revealed a 4-5Hz theta activity, ample, symmetrical, bilateral, and associated with epileptogenic graphoelements of diffuse point type accentuated in the right hemisphere, suggesting multifocal epilepsy (Figure 1). The COVID-19 polymerase chain reaction (PCR) confirmatory test that was performed at admission was later (24h) positive and the D-dimer level was 2340μg/L. The patient was transferred to the national COVID-19 case management center and subjected to chloroquine sulfate 500mg/day. This molecule was later discontinued on the neurologist's recommendation due to the paroxysmal recurrence of seizures. Several other tracings were requested but were not performed due to the non-availability of EEG in the said structure. He later received corticosteroid therapy and the rest of the management was symptomatic. After 1 month without seizures, following 2 reverse transcription-polymerase chain reaction (RT-PCR) tests, the patient was discharged with a prescription issued by the Neurologist.

**Case N°2:** patient aged 11 years, followed at the National Center for the Management of Persons Infected with COVID-19 for influenza-like illness, respiratory distress and confirmed positive under treatment with chloroquine sulfate 500mg/day, azythromycin 250mg/day. After 1 week of treatment, loss of consciousness with generalized tonic-clonic seizure, lateral biting of the tongue, hyper salivation and loss of urine occurs. There is no personal or family history of epileptic seizures. According to the mother, the delivery took place in better condition. The clinical examination found respiratory difficulty, tachycardia at 123beats/minute but apyretic. The neurological examination in the intercritical phase found persistent mental confusion for more than 1 hour. The metabolic balance sheet is within the limits of normal, the other biological parameters reveal a non-specific inflammatory syndrome. The electrocardiogram (ECG) showed a heart rhythm disorder. Brain CT scan and intercranial EEG were not performed urgently. We recommended discontinuation of chloroquine sulfate and initiation of clonazepam 1ml intravenous (IV) Benzodiazepam as needed. Background antiepileptic [Levitracetam (keppra 500g/day)] was instituted following the second seizure. After recovery from the COVID-19 infection, and the absence of seizure for 3 weeks following the new treatment recommendations, we performed a brain scan and EEG, which were found to be normal. The patient was discharged and recommended for neurological surveillance.

**Case N°3:** patient aged 16 years, followed at the national center for the management of COVID-19 positive cases. Patient was asymptomatic on admission. No known drug defects or allergies. Following a voluntary RT-PCR test that the patient performed, he was diagnosed positive for COVID-19 and started treatment with chloroquine sulfate 500mg/day, Azythromycin 250mg/day. After 4 days of treatment, a loss of consciousness with Bravais Jacksonian clonic seizures of the right hemicorps occurred. No personal or family history of epileptic seizures is found. The clinical examination does not reveal any particularities. There was no postcritical confusion and the rest of the neurological examination was normal. The diagnosis of focal epilepsy with roladic paroxysm was made. The metabolic assessment was within normal limits, the other biological parameters revealed a non-specific inflammatory syndrome. We recommended discontinuation of chloroquine sulfate and initiation of clonazepam 1ml IV benzodiazepine as needed. Levitracetam 500g/day was instituted following a repeat attack within 48 hours after discontinuation of chloroquine. The patient was discharged with 16 days of seizure-free follow-up and 2 negative RT-PCR tests. Brain CT scan and EEG performed after recovery showed no detectable abnormalities.

## Discussion

Seizures have not been reported as a direct manifestation of SARS-CoV-2 infection. During the duration of this study, out of 365 children under 16 years of age diagnosed positive for COVID-19, only 1 case (3.65%) was insidiously diagnosed in the etiological investigation of an epileptic seizure and 2 (7.3%) cases presented with an epileptic seizure after initiation of chloroquine therapy (Table 1).

**Table 1:** distribution of patients according to clinical and paraclinical and therapeutic observations

Observations	Case 1	Case 2	Case 3
Clinical/paraclinical/therapeutic			
Age (years)	14	11	16
Gender	Men	Men	Men
Notion of patient contact COVID-19	No	Unknown	Unknown
Admitted for epileptic seizures	Yes	No	No
Recent influenza syndrome	Yes	Yes	Yes
Known epileptic patient - compliance with antiepileptic treatment - occurrence of the crisis in an infectious context	Yes	No	No
	Yes		
	Yes		
Symptomatic to COVID-19	Yes	Yes	Yes
Nucleic acid test by real-time PCR	Positive	Positive	Positive
CSF-PCR	Not reallized	Not reallized	Not reallized
Brain CT	Normal	Not reallized	Not reallized
EEG	Pathological	Not reallized	Not reallized
Occurrence of seizures before taking chloroquine sulfate	Yes	No	No
Occurrence or aggravation of seizures after taking chloroquine sulfate	Yes	Yes	Yes
Anti-epileptic drugs used	Clonazepam Phenobarbital Thiopental Sodium valproate	Clonazepam Levitracetam	Clonazepam Levitracetam
Evolution	Cured of COVID-19 and stop seizures	Cured of COVID-19 and stop seizures	Cured of COVID-19 and stop seizures

Given that the first patient was previously known to have had an epileptic seizure, a new seizure is hardly surprising. On the other hand, the sudden onset of a paroxysmal seizure with a generalized recurrence quickly attracts our attention. According to Baig AM *et al*. once the virus penetrates the blood-brain barrier, it can slow brain microcirculation to promote increased interaction of SARS-CoV-2 with endothelial and glial tissue receptors [5]. This predisposes patients to epileptic seizures, as noted in the experience of Newey CR and colleagues [7]. Similarly, in our case, the absence of brain damage visible on imaging suggests a biochemical mechanism following the rupture of the blood-brain barrier with accumulation of inflammatory markers. This could result in local cortical irritation that would precipitate the seizures associated with COVID-19 infection. Although cerebrospinal fluid may contain markers of inflammation justifying cerebral aggression, we were unable to perform the cerebrospinal fluid-polymerase chain reaction (CSF-PCR) assay for SARS-CoV-2 in our patients. We believe that epileptic seizures may be a manifestation of COVID-19 disease in the CNS. Although no direct evidence of a neurotropic effect of SARS-CoV-2 has yet been reported, either from cerebrospinal fluid (CSF) studies or autopsies, the presence of neurological symptoms in patients with COVID-19 disease during the current pandemic and the similarity between the two human coronavirus strains (CoV-1 and CoV-2) make this mechanism highly suggestive. Given the rapid progression of SARS-CoV-2 infection and the desperate need for a drastic measure to eradicate the disease, we are making therapeutic choices that are favorable for some patients but cause neurotransmission dysfunction for others. All the children presented in this work initially benefited from treatment with chloroquine Sulfate at a dose of 500mg/day before being stopped later because of worsening seizures. According to Hassanipour *et al*. chloroquine at 10-50mg/kg has a pro-convulsive effect by inhibiting GABAergic neurotransmission [6]. These experiments suggest that chloroquine is seizure inducing in some individuals, as 10.95% of the study population had a seizure within 7 days. However, there is insufficient data and evidence to support this probability. It should also be noted that 89.05% of the children who received the same drug did not have a seizure during the entire hospitalization period. Although randomized studies do not yet support the efficacy of chloroquine Sulfate in the management of COVID-19, we have reason to believe in its usefulness in this context where there is no other effective therapeutic alternative. On the other hand, its interest in epileptic children remains to be verified by means of a large-scale study. The absence of EEG in COVID-19 patient management facilities was the main limitation of this study. Further research is needed to elucidate the mechanism of neurological symptoms in SARS-CoV-2 infection and the involvement of chloroquine sulfate in epileptogenesis.

## Conclusion

In order to better understand the subject, it is now necessary to conduct cohort studies linking the involvement of SARS-CoV-2 in the aggravation of seizures in known epileptic children and in the epileptogenesis of children hitherto seizure-free. The search for direct evidence of a neurotropic effect of SARS-CoV-2 on cerebrospinal fluid (CSF) or through autopsies appears to be the best option. Finally, equipping COVID-19 patient management centers with EEGs could facilitate diagnosis and continuous electroencephalographic monitoring, especially in cases of epileptic seizure disorders.
